# Pennsylvania State Board of Veterinary Medical Examiners

**Published:** 1895-12

**Authors:** 


### PENNSYLVANIA STATE BOARD OF VETERINARY MEDICAL EXAMINERS.

The State Board of Veterinary Medical Examiners of Pennsylvania were convened at Harrisburg at 12 M. on November 13th, in accordance with a call issued by the Secretary of the Commonwealth. The Board being duly sworn into office by the Department of State, Dr. W. Horace Hoskins was made temporary Chairman, and Dr. S. J. J. Harger, Secretary *pro tem.* All the members of the Board were present.

First order of business being election of permanent officers, Dr. S. J. J. Harger was nominated for President by Dr. Harry Walters, and Dr. W. Horace Hoskins for Secretary by Dr. J. C. McNeil. No other names being placed in nomination, the election was made by acclamation, upon which the officers assumed their respective places.

The question of adopting suitable by-laws for the government of the Board was taken up for consideration, and after considerable discussion on the subject a motion was made that the Chair appoint a committee of three to prepare suitable rules to be submitted to the entire Board for adoption at the next meeting, and that they also draft a suitable form of license to be presented at the same time.

The Secretary was instructed, on motion, to procure a minute-book and a suitable registration book.

Deputy-Secretary Barnet was then asked to inform the Board as to the intent of the act so far as placing on the seal of the State, and the Board were informed that all licenses granted would have to be returned to the Department of State at Harrisburg to receive there the seal of the Commonwealth.

It was also suggested to the Committee on Rules and By-laws that the officers be elected annually. It was also decided that two regular meetings be held each year, in the latter part of April and June, with such other meetings as the business of the Board might demand.

The subjects for examination were then assigned as follows: Anatomy, veterinary diagnosis, and zoötechnics, by Dr. S. J. J. Harger; practice of medicine, by Dr. W. Horace Hoskins; obstetrics, meat- and milk-inspection, and sanitary medicine, by Dr. J. W. Sallade; surgery and physiology, by Dr. Harry Walters; pathology, chemistry, materia medica, and therapeutics, by Dr. J. C. McNeil. The consideration of the age of the applicants, their moral character, and genuineness of their diplomas, was placed in the hands of the Secretary.

It was also decided that a list of fifteen questions on each branch would be submitted to the applicants, from which they shall select ten for answering. Applicant shall draw one from a box of cards with numbers upon them, upon which he shall write his name, and place the same in a sealed envelope, and shall designate his answers to the questions on the various subjects by the same number. This card shall be placed in the keeping of the Secretary. It was decided that a general average of 65 per cent. shall have been attained, and that a minimum of 50 per cent. must be attained on each branch.

It was moved and seconded that the April meeting shall convene for two days. It was decided that the first regular meeting of the Board for examination of applicants for license shall convene on the third Monday in December, at the office of the

Secretary, 3452 Ludlow Street, Philadelphia, Pa., and that notices of the same shall be sent to the veterinary journals of North America.

The Chairman then appointed the following Committee on Rules and By-laws: Dr. W. Horace Hoskins, Chairman; Dr. J. W. Sallade, and Dr. Harry Walters; after which the motion to adjourn prevailed.

W. HORACE HOSKINS, Secretary, 3452 Ludlow Street, Philadelphia.

S. J. J. HARGER, President.

